# Supplementary material for: An optimum rate of microtubule flux for error correction in metaphase spindle
Source: Life Sci Alliance. 2026 Apr 27;9(7):e202503612. doi: 10.26508/lsa.202503612 (PMC13121783; doi:10.26508/lsa.202503612)
Supplement: Supplementary file 1 [file LSA-2025-03612_TableS1.doc]

**Table S1. Parameter values of kinesin-5 Eg5 motor**

| Parameter | Value | Source |
| --- | --- | --- |
| *k*^(+)^ ($\text{s}^{-1}$) | 25.6 | Liu et al., 2021; Wang et al., 2025 |
| *k*^(−)^ ($\text{s}^{-1}$) | *k*^(+)^/15 | Liu et al., 2021 |
| *E*_D_ (*k*_B_*T*) | 5 | Liu et al., 2021 |
| *k*_NL_ ($\text{s}^{-1}$) | 400 | Liu et al., 2021 |
| *k*_D_ ($\text{s}^{-1}$) | 100 | Liu et al., 2021; Wang et al., 2025 |
| *k*_r_ ($\text{s}^{-1}$) | 9.2 | Liu et al., 2021 |
| $\delta_{\text{w}}$ (nm) | 1 | Liu et al., 2021 |
| $\varepsilon_{\text{w0}}$ ($\text{s}^{-1}$) | 5 | Liu et al., 2021 |
| $\delta_{\text{s}}$ (nm) | 2.3 | Wang et al., 2025 |
| $\varepsilon_{\text{s0}}$ ($\text{s}^{-1}$) | 0.1 | Wang et al., 2025 |
| *μ*_m_ ($\text{s}^{-1}$) | 0.2 | Wang et al., 2025 |
| $k_{\text{on}}^{\left(\text{m}\right)}$($\text{nM}^{-1}\text{s}^{-1}\text{site}^{-1}$) | $4\times10^{-4}$ | Wang et al., 2025 |
| *K*_E_ (pN/nm) | 0.55 | Wang et al., 2025 |
| [K5] (nM) | 3 | Wang et al., 2025 |

As defined in Liu et al. (2021) and Wang et al. (2025), *k*^(+)^ is the rate of ATP transition to ADP in the head with its neck linker (NL) in the plus-ended orientation (e.g., the trailing head), *k*^(−)^ is the rate of ATP transition to ADP in the head with its NL not in the plus-ended orientation (e.g., the leading head), *E*_D_ is the energy change associated with the conformational change of the head and NL docking induced by ATP binding, *k*_NL_ is the rate of NL docking, *k*_D_ is the rate of ADP release from the head bound to MT, *k*_r_ is the rate of the tail domain releasing from the nucleotide-free head, $\varepsilon_{\text{w0}}$ is the dissociation rate of the motor under no load during the weak-MT binding state with affinity *E*_w2_, $\delta_{\text{w}}$ is the load-sensitivity distance for the dissociation during the weak-MT binding state, $\varepsilon_{\text{s0}}$ is the dissociation rate of the motor under no load during the strong-MT binding state, $\delta_{\text{s}}$ is the load-sensitivity distance for the dissociation during the strong-MT binding state, $\mu_{\text{m}}$ is the binding rate of one pair of the heads to one MT when another pair of the heads at the opposite end of the stalk are attached to another MT in the antiparallel overlap zone, $k_{\text{on}}^{\left(\text{m}\right)}$ is the second-order MT-binding rate of the kinesin-5 motor in solution, [K5] is kinesin-5 concentration, and *K*_E_ is the elastic constant of the motor’s stalk.
