# Supplementary material for: An optimum rate of microtubule flux for error correction in metaphase spindle
Source: Life Sci Alliance. 2026 Apr 27;9(7):e202503612. doi: 10.26508/lsa.202503612 (PMC13121783; doi:10.26508/lsa.202503612)
Supplement: Supplementary file 2 [file LSA-2025-03612_TableS2.doc]

**Table S2. Parameter values of kinesin-13 motor**

| Parameter | Value | Source |
| --- | --- | --- |
| $k_{\text{dep}}$(s^-1^) | 5 | Xie, 2024 |
| $\tau_{\text{end}}$ (s) | 2 | Wang et al., 2025 |
| $k_{\text{diff}}^{\text{(K13)}}$ (s^-1^) | 82 | Wang et al., 2025 |
| [K13] (nM) | 1 | Wang et al., 2025 |
| $k_{\text{on}}^{\left(\text{K13}\right)}$(nM^-1^s^-1^nm^-1^) | $3\times10^{-4}$ | Wang et al., 2025 |
| $k_{\text{off}}^{\left(\text{K13}\right)}$ (s^-1^) | $3\times10^{-3}$ | Wang et al., 2025 |

As defined in Wang et al. (2025), $k_{\text{dep}}$ is the depolymerization rates of the kinesin-13 motor at the minus end, $\tau_{\text{end}}$ is the MT-end residence time of the kinesin-13 motor, $k_{\text{diff}}^{\text{(K13)}}$ is the forward or backward stepping rate of kinesin-13 along MT due to diffusion, $k_{\text{off}}^{\left(\text{K13}\right)}$ is the detachment rate of kinesin-13 from MT, $k_{\text{on}}^{\left(\text{K13}\right)}$ is the second-order binding rate of kinesin-13 to MT and [K13] is kinesin-13 concentration.
