# Supplementary material for: An optimum rate of microtubule flux for error correction in metaphase spindle
Source: Life Sci Alliance. 2026 Apr 27;9(7):e202503612. doi: 10.26508/lsa.202503612 (PMC13121783; doi:10.26508/lsa.202503612)
Supplement: Supplementary file 3 [file LSA-2025-03612_TableS3.doc]

**Table S3. Parameter values of NuMA protein**

| Parameter | Value | Source |
| --- | --- | --- |
| $k_{\text{on}}^{\left(\text{NuMA}\right)}$ (nM^-1^ s^-1^site^-1^) | $3\times10^{-4}$ | Wang et al., 2025 |
| [NuMA] (nM) | 1 | Wang et al., 2025 |
| $K_{\text{NuMA}}$ (pN/nm) | 0.03 | Wang et al., 2025 |
| $\mu_{\text{NuMA}}$ (s^-1^) | 1 | Wang et al., 2025 |

As defined in Wang et al. (2025), $k_{\text{on}}^{\left(\text{NuMA}\right)}$ is the second-order binding rate of NuMA to MT, [NuMA] is the NuMA concentration, $\mu_{\text{NuMA}}$ is the binding rate of one head to MT when another head at the opposite end of the stalk is attached to another parallel MT, and $K_{\text{NuMA}}$ is the elastic constant of the NuMA stalk.
