# Supplementary material for: An optimum rate of microtubule flux for error correction in metaphase spindle
Source: Life Sci Alliance. 2026 Apr 27;9(7):e202503612. doi: 10.26508/lsa.202503612 (PMC13121783; doi:10.26508/lsa.202503612)
Supplement: Supplementary file 4 [file LSA-2025-03612_TableS4.doc]

**Table S4. Parameter values related to the polymerization of kMT from the plus end**

| Parameter | Value | Source |
| --- | --- | --- |
| *B* | 4 | Wang et al., 2025 |
| *F*_p0_ (pN) | 1.85 | Wang et al., 2025 |
