# Supplementary material for: An optimum rate of microtubule flux for error correction in metaphase spindle
Source: Life Sci Alliance. 2026 Apr 27;9(7):e202503612. doi: 10.26508/lsa.202503612 (PMC13121783; doi:10.26508/lsa.202503612)
Supplement: Supplementary file 5 [file LSA-2025-03612_TableS5.doc]

**Table S5. Parameter values of spring elastic constants**

| Parameter | Parameter description | Value | Source |
| --- | --- | --- | --- |
| $\kappa$ (pN/nm) | $N\kappa$is the elastic constant of spring connecting two kinetochores | 0.01 | Wang et al., 2025 |
| $\kappa_{2}$ (pN/nm) | $\kappa_{2}$ is the elastic constant of spring connecting kinetochore and plus end of each kMT | 0.1 | Wang et al., 2025 |
| $\kappa_{3}$ (pN/nm) | $\kappa_{3}$ is the elastic constant of spring connecting pole and minus end of each MT | 0.1 | Wang et al., 2025 |
